# Structural insights into DNA recognition by AimR of the arbitrium communication system in the SPbeta phage

**DOI:** 10.1038/s41421-019-0101-2

**Published:** 2019-05-28

**Authors:** Zeyuan Guan, Kai Pei, Jing Wang, Yongqing Cui, Xiang Zhu, Xiang Su, Yuanbao Zhou, Delin Zhang, Chun Tang, Ping Yin, Zhu Liu, Tingting Zou

**Affiliations:** 1 National Key Laboratory of Crop Genetic Improvement and National Centre of Plant Gene Research, Huazhong Agricultural University, Wuhan, 430070 Hubei China; 2 CAS Key Laboratory of Magnetic Resonance in Biological Systems, State Key Laboratory of Magnetic Resonance and Atomic Molecular Physics, and National Center for Magnetic Resonance at Wuhan, Wuhan Institute of Physics and Mathematics, Chinese Academy of Sciences, Wuhan, 430071 Hubei Province China; 3 College of Life Science and Technology, Huazhong Agricultural University, Wuhan, 430070 Hubei China

**Keywords:** X-ray crystallography, Transcription

## Abstract

A newly identified arbitrium communication system regulates the lysis-to-lysogeny decision in a *Bacillus* bacteriophage. This system contains an arbitrium hexapeptide as a signal, the cellular receptor AimR, and the lysogenic negative regulator AimX. AimR specifically targets the downstream DNA to activate *aimX* gene expression. The arbitrium peptide binds to AimR, inhibiting its DNA-binding to promote phage lysogeny. Recently, we and other groups have elucidated how arbitrium peptide sensed by AimR. However, the molecular mechanisms of DNA recognition by AimR and the regulation of its DNA-binding activity by the peptide remain largely unknown. Here, we report the crystal structure of the AimR–DNA complex at 2.1 Å resolution. The N-terminal HTH motif recognizes the palindromic DNA sequence, buttressed by interactions between positively charged residues and the DNA phosphate groups. The DNA-bound AimR assembles a more closed dimer than the peptide-bound form. Single-molecule FRET and crosslinking assays revealed that the AimR protein samples both open and closed conformations in solution. Arbitrium peptide binding induces a closed-to-open conformational change of AimR, eliminating DNA targeting. Our structural and functional analysis provides new insights into the DNA recognition mechanism of AimR and its regulation by the arbitrium peptide in the context of phage lysis-lysogeny decisions.

## Introduction

Gram-positive bacteria communicate with each other via secreted peptide signals to adapt to the environment by regulating gene expression related to bacterial sporulation, biofilm formation, the virulence activation process, etc.^1–7^. Typically, 5- to 10-amino acid signal peptides are sensed by receptors, leading to allosteric regulation of the receptors’ activity. In the quorum-sensing system, the receptors are Rap-Rgg-NprR-PlcR-PrgX (RRNPP) members, which employ an N-terminal domain to bind to target DNA or partner proteins and a C-terminal tetratricopeptide repeat (TPR) domain to coordinate the peptide. The molecular mechanisms by which peptides regulate the activities of RRNPP proteins are well studied^8–10^.

Recently, in *Bacillus* phages, a peptide communication system named the arbitrium system was identified that plays an essential role in lysis–lysogeny decisions during infection of *Bacillus* host cells^11–14^. This system requires the arbitrium hexapeptide to bind to its cellular receptor AimR to regulate transcription of the lysogeny negative regulator gene *aimX*. After infecting *Bacillus* host cells, the phage produces the precursors of signal peptides encoded by the *aimP* gene. The premature peptides are secreted to the extracellular environment and cleaved by bacterial extracellular proteases to generate the mature hexapeptide. The secreted peptides are imported into the host cell through an oligopeptide permease transporter and then bind to the AimR receptor, which functions as a transcription factor. Consequently, peptide-bound AimR loses its DNA-binding activity. This signal enables phage integration into the *Bacillus* genome as a prophage^11^.

AimR is composed of two domains, a N-terminal domain that is responsible for DNA binding and a C-terminal domain that specifically recognizes the arbitrium peptide. In a previous study, we reported the structures of AimR with and without the arbitrium peptide from SPbeta group phages^15^. The overall structure of AimR is similar to that of RRNPP family proteins. The C-terminal domain coordinates the arbitrium peptide GMPRGA via its four atypical TPR repeats. Furthermore, we biochemically demonstrated that AimR specifically targets a 51-bp DNA sequence downstream of the *aimP* gene, and its DNA-binding activity is decreased upon peptide binding. Interestingly, both the apo form and peptide-bound form of AimR exhibit dimer formation mediated through the last C-terminal capping helix. Only subtle conformational changes were observed between apo- and peptide-bound AimR. Similar results were obtained by other groups^16,17^. Despite this progress, it remains unknown whether DNA-bound AimR adopts a distinct conformation, whether the peptide inhibits these conformational changes and, if so, how this might happen.

Here, we report the complex structure of the AimR protein and cognate DNA. Dimeric AimR binds to a palindromic DNA via two interaction areas. The N terminus of AimR exhibits a classic HTH motif that specifically recognizes a trinucleotide inverted repeat. In addition, a patch of positively charged residues from the C-terminal TPR motifs coordinate the DNA phosphate groups to reinforce the interactions. Substitutions of the key DNA recognition residues are unable to initiate phage lysogeny in vivo. Compared with the structures of the apo form and peptide-bound form, the conformation of DNA-bound AimR becomes more closed, generating a second dimer interface. Furthermore, by single-molecule fluorescence resonance energy transfer (smFRET) and crosslinking assays, we found that in solution, the AimR protein samples two conformational states, a closed state and an open state. In the presence of the arbitrium peptide, the population of the open state is increased, while upon binding to DNA, the majority of AimR remains in the closed state, in line with the structural observations. Taken together, the crystal structures and dynamic structural studies not only reveal the molecular mechanism by which the arbitrium peptide regulates the DNA-binding activity of the AimR protein from the SPbeta phage in lysis–lysogeny decisions but also serve as a framework for investigating the ligand regulation functions of proteins in the RRNPP family.

## Results

### Crystal structure of the SPbeta AimR–DNA complex

**Fig. 1:**
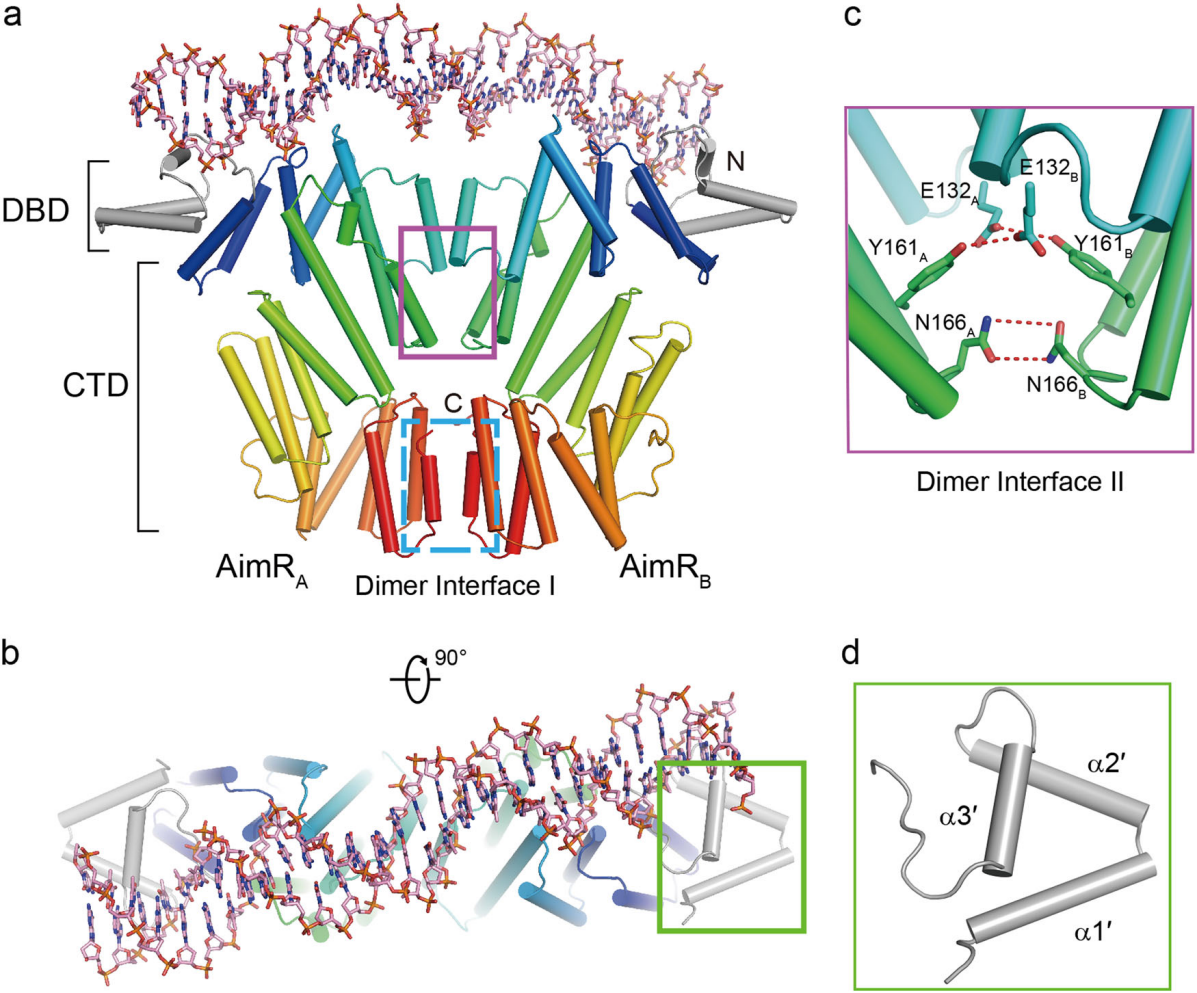
Structure of AimR–DNA complex. **a**, **b** The overall structure of AimR–DNA. The CTDs of AimR are rainbow-colored and the DBDs of AimR are colored in gray. The DNA fragment (5′ CTTAAATATTAGGTTTTAATAACATCTAGT 3′) is shown by stick. Two dimer interfaces between the two protomers of AimR are highlighted using rectangle. **c** Close-up view of dimer interface II. The hydrogen bonds are represented by red dotted lines. **d** The α-helices are sequentially numbered from the N terminus of the DBD

To understand how AimR recognizes the DNA sequence, we reconstituted and crystallized a binary complex between the AimR protein and the cognate DNA fragment from the SPbeta group phage. Previously, we identified a 51 bp DNA segment (locus 77704–77754 downstream of the *aimP* gene in the SPbeta phage) that binds the AimR protein with a dissociation constant (*K*_d_) of 101 nM through electrophoretic mobility shift assays (EMSA)^15^. The DNA fragment contains a 31 bp palindromic sequence (5′ ACTTAAATATTAGGTTTTAATAACATCTAGT 3′, locus 77710–77740 in the SPbeta phage genome) (Supplementary Fig. S1). The 31 bp fragment retains the ability to bind AimR (see results below). Deletion of one or more nucleotides results in a notably decreased dissociation constant. After numerous crystallization trials for AimR with different DNA fragments, we finally determined the structure of full-length AimR in the presence of the 31 bp DNA at 2.10 Å resolution by molecular replacement. In the crystals, there is one AimR dimer in each asymmetrical unit. Most amino acids and nucleotides are assigned to the electron density map. Each AimR protomer consists of an N-terminal DNA-binding domain (DBD, residues 1–48) and a C-terminal regulatory domain (CTD, residues 49–386). The DBD is a typical HTH DNA-binding motif containing a three-helix bundle (α1′, α2′, and α3′) (Fig. 1a, d), which is conserved among the *Bacillus* phages (Supplementary Fig. S2). The CTD is composed of 20 α-helices that form a right-handed superhelical assembly. In the apo- and peptide-bound form, the AimR dimer exhibits an inverted trapezoid-like fold. The last C-terminal capping helices from two AimR molecules undergo dimerization via a network of van der Waals interactions. In contrast, the DNA-bound AimR dimer has two interaction faces (Fig. 1a). One face is identical to that of the apo- and peptide-bound form. The other face is generated by the residues Glu132, Tyr161, and Asn166 from helix 6, helix 7, and loop 7, respectively, of each protomer. These residues form hydrogen bonds with each other (Fig. 1c).

### DNA recognition mode

**Fig. 2:**
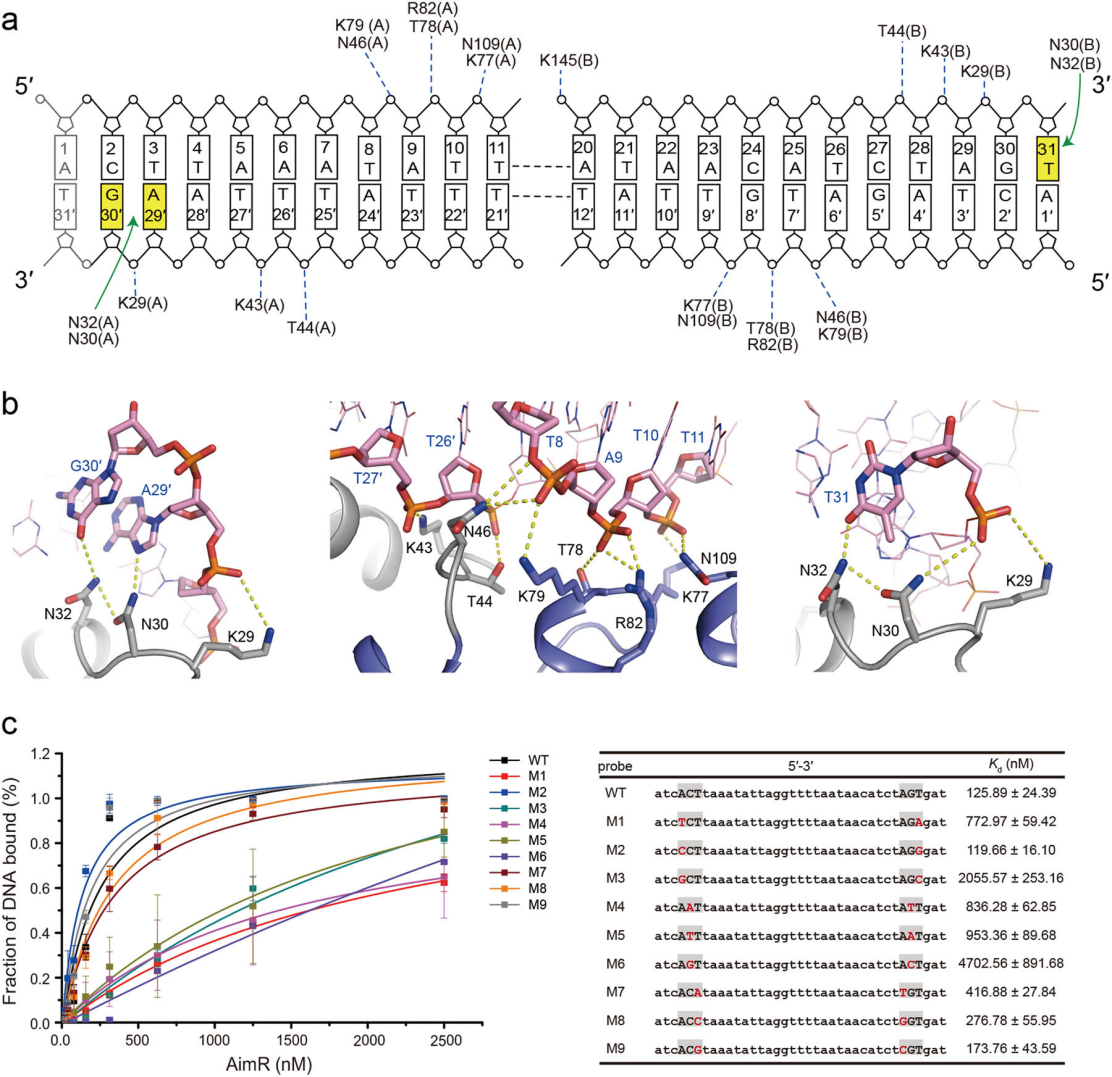
DNA recognition by the AimR protein. **a** Schematic view of AimR–DNA contacts. The bases are labeled according to the symmetry of the DNA sequence. Hydrophobic contacts are shown as green arrows, while nonspecific interactions are indicated by blue dotted lines. **b** Key residues and nucleotides involved in the interactions. Protein side chains and DNA bases are shown in a stick representation. DNA phosphate backbones and bases are colored orange and pink, respectively. **c** Examination of the DNA-binding affinity of AimR toward various DNA. The DNA sequence of each probe and the respective *K*_d_ values are listed in the table. The varied nucleotides are highlighted in red. Error bars indicate the s.d. of at least three independent measurements (the points are the mean)

The interaction between AimR and DNA is primarily mediated by the N-terminal area, including extensive hydrogen bonds and ionic interactions, giving rise to two major interfaces (approximately 1300 Å, Fig. 1). First, each protomer of the AimR dimer symmetrically binds to the major grooves of the DNA fragment through its DBD domain (Fig. 1a). The recognition helix α3′ from each DBD domain is oriented to the DNA axis and specifically interacts with bases from the major groove via the side chains of residues Asn30 and Asn32 (Fig. 2b). Considering protomer A, the side chains of Asn30 and Asn32 form hydrogen bonds with base A29′ and G30′ on the antisense strand (Fig. 2a, b). The side chains of Asn30 and Asn32 from protomer B establish base-specific interactions with base T31 on the sense strand (Fig. 2a, b). Buttressing the hydrogen bonds, ten residues (Lys29, Lys43, Thr44, Asn46, Lys77, Thr78, Lys79, Arg82, Asn109, and Lys145) in the positively charged patches contribute coordination of the backbone phosphates of T9, T10, T11, A20, T28, A29, G30, T7′, G8′, T9′, T26′, T27′, and A29′, constituting the second interface (Fig. 2a, b). These structural observations are consistent with our previous biochemical analysis^15^. The whole DNA fragment displays the characteristic shape of a right-handed B-form nucleic acid structure with average global roll and twist angles of −1.14° and 36.09°, respectively. Whereas the major groove width at the recognition site is widened to 18.54 Å, and the average minor groove width is narrowed to 10.35 Å, demonstrating the conformational changes of DNA upon AimR binding (Supplementary Fig. S3).

To verify the base-recognition specificity observed in the AimR–DNA complex, we mutated the palindromic sequence (5′ ACT……AGT 3′) one by one to assess the binding affinities of AimR (Fig. 2c and Supplementary Fig. S4). AimR binds to DNA with an apparent dissociation constant (*K*_d_) of 100.5 ± 27.6 nM, consistent with our previous study^15^. The most significant effect of base substitution was observed at C2 (G30′), and mutation of C2 to adenine, guanine, and thymine reduced the AimR DNA binding activities by approximately 8-, 9-, and 42-fold, respectively. A1T/T31′A and A1G/T31′C substitution lowered the AimR-binding affinity by approximately 7- and 19-fold, respectively, while other mutations had little influence on the AimR–DNA interaction (Supplementary Fig. S4). Together with the structural observations, the DNA mutational analysis suggests that the adenine and cytosine bases at the palindromic repeats are essential for DNA recognition by AimR.

### Functional implications of the DNA-binding residues of AimR

**Fig. 3:**
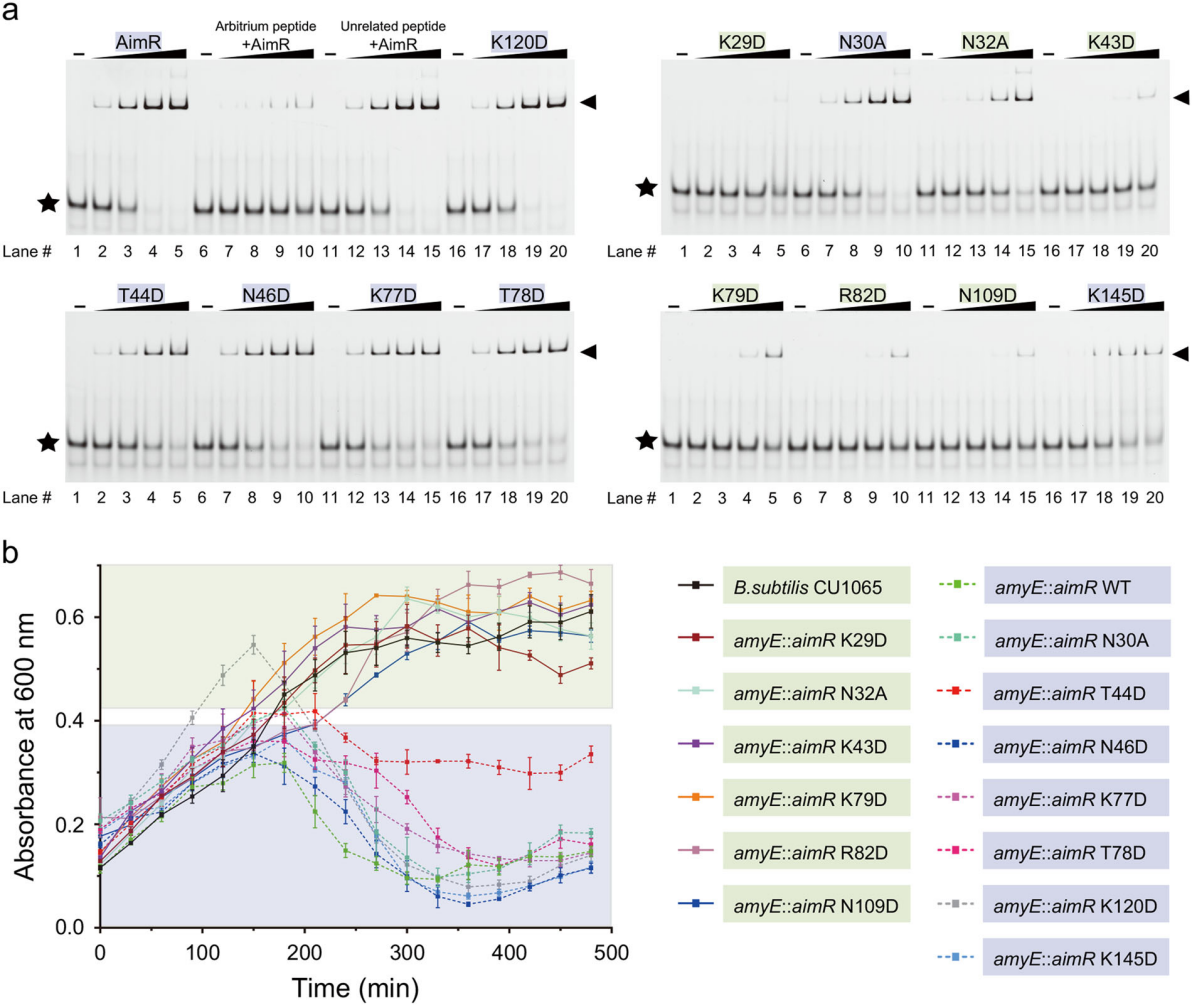
Effect of AimR mutations on DNA binding and infection dynamics of SPbeta phage. **a** DNA-binding activities of wild-type AimR and AimR mutants were examined. The AimR concentrations in lanes 1–5 were 0, 0.008, 0.04, 0.2, and 1 μM, respectively. The concentration of the 31 bp DNA fragment was 20 nM. The arbitrium peptide (GMPRGA) and an unrelated peptide (GFGHGA) were added as controls. AimR–DNA complex is indicated by a black arrowhead, and free DNA is indicated by a black asterisk. **b** Effect of AimR mutations on the infection dynamics of SPbeta. Growth curves of *B. subtilis* strain CU1065 infected with SPbeta, or that of *B. subtilis* strain CU1065 (*amyE*::*aimR* WT, K29D, N30A, N32A, K43D, T44D, N46D, K77D, T78D, K79D, R82D, N109D, K145D, or K120D) infected with SPbeta Δ*aimR* at MOI = 0.001. Data represent an average of three biological replicates, and each with three technical replicates. Error bars indicate the s.d. of at least three independent measurements (the points are the mean)

The importance of residues involved in DNA recognition is supported by mutational analysis. As anticipated, AimR binds to its cognate DNA; the arbitrium peptide disrupts this interaction, but the unrelated control peptide does not (Fig. 3a, left upper panel). Several mutants, K29D, N32A, K43D, K79D, R82D, N109D, and K145D, show notable weak DNA-binding activity, but N30A, T44D, N46D, K77D, and T78D retain activity comparable to that of the control (Fig. 3a and Supplementary Fig. S5). As a control, we also mutated the unrelated Lys120 to Asp, with no detectable effects on DNA binding (Fig. 3a, left upper panel). All of these mutant proteins exhibited behavior similar to that of wild-type AimR via gel filtration (Supplementary Fig. S6). Moreover, we measured the DNA-binding affinities of these AimR mutations (Supplementary Fig. S5). AimR N32A showed a significant decrease in DNA-binding activity, with *K*_d_ = 6824.02 ± 2250.58 nM. In addition, T44D reduced the DNA-binding affinity of AimR by 11-fold, but four mutations (N30A, N46D K77D, and T78D) had little impact on DNA targeting (Supplementary Fig. S5).

We further assessed the effect of DNA-binding mutations on the lysis–lysogeny decision during SPbeta phage infection. First, we constructed the *aimR* deletion SPbeta phage (Δ*aimR*) to examine the infection dynamics in the host bacteria (*Bacillus subtilis* CU1065) or *aimR*-gene-recovered cells. After infection with SPbeta Δ*aimR*, the bacteria continued to grow (Fig. 3b, black curve), but the cells that overexpressed the AimR protein (*B. subtilis* CU1065, *amyE*::*aimR*) lysed within 3 h (light green curve). Next, we prepared a dozen strains that integrated *aimR* mutant genes into the host genome. The mutants with DNA-binding affinities similar to those of the wild-type AimR protein had similar lysis curves, except for K145D. However, no induced lysis was observed in *B. subtilis* CU1065 (*amyE*::*aimR* K29D, N32A, K43D, K79D, R82D, or N109D) (Fig. 3b). Overall, structural and functional analysis demonstrates that these residues play an essential role in DNA binding.

### The DNA-bound AimR dimer exhibits a closed conformation

In our previous reported structures of apo- and peptide-bound AimR, we were unable to trace the N-terminal DBD structure due to the poor electronic densities^15^. In this study, we reoptimized the crystals and collected new datasets for AimR with or without the peptide. Crystal structure determination was performed by molecular replacement using the DNA-bound DBD of AimR as the template. Finally, we traced the density of the DBD for AimR with or without the peptide (Supplementary Fig. S7 and Table 1). Although both the apo- and peptide-bound AimR structures exhibited nearly identical structures with an average root mean square deviation of 1.459 Å over an average of 776 aligned Cα atoms, the DBD of apo- or peptide-bound AimR exhibited a more flexible conformation than the DNA-bound AimR (Supplementary Fig. S7a–c).

**Table 1:** Statistics of data collection and refinement

Structure	AimR (PDB: 6JG5)	AimR–peptide (PDB: 6JG9)	AimR–DNA (PDB: 6JG8)
Data collection			
Space group	*P2*_*1*_ *2*_*1*_ *2*	*P2*_*1*_ *2*_*1*_ *2*	*P4*_*1*_
Unit cell dimensions			
*a*, *b*, *c* (Å)	33.49, 115.30, 219.43	33.59,120.93, 214.0	98.01, 98.01, 159.99
*α*, *β*, *γ* (°)	90, 90, 90	90, 90, 90	90, 90, 90
Wavelength (Å)	0.9792	0.9778	0.9792
Resolution (Å)	45–2.22 (2.29–2.22)	45–2.0 (2.05–2.0)	45–2.1 (2.13–2.10)
*R*_merge_ (%)	10.9 (34.1)	7.1 (38.9)	4.1 (68.9)
*I*/*σ*(*I*)	10.9 (4.9)	17.8 (4.2)	23.4 (3.1)
Completeness (%)	99.1 (96.0)	99.8 (96.2)	99.7 (98.5)
Number of measured reflections	276,186	396,286	600,917
Number of unique reflections	42,901	60,685	87,895
Redundancy	6.4 (6.2)	6.5 (6.2)	6.8 (7.0)
Wilson *B* factor (Å^2^)	24.9	23.8	44.4
Refinement			
*R*_work_/*R*_free_ (%)	19.62/23.94	21.35/24.88	21.06/22.30
Number of atoms			
Protein main chain	3120	3116	3116
Protein side chain	3304	3279	3313
Protein all atoms	6424	6395	6429
Water molecules	329	406	257
Other entities	0	80	1227
All atoms	6753	6872	7913
Average *B* value (Å^2^)			
Protein main chain	36.8	42.1	54.8
Protein side chain	41.6	46.0	62.4
Protein all atoms	39.2	44.1	58.7
Water molecules	39.8	25.4	57.3
Other entities	0	42.9	79.4
All atoms	39.3	43.8	61.9
R.M.S. deviations from ideal values			
Bonds (Å)	0.009	0.010	0.009
Angle (°)	0.956	1.094	1.067
Ramachandran plot statistics (%)			
Most favorable	93.2	94.4	92.1
Additionally allowed	6.8	5.6	7.8
Generously allowed	0	0	0.1
Disallowed	0	0	0

Values in parentheses are for the highest resolution shell. *R*_merge_ = Σ_*h*_Σ_*i*_|*I*_*h,i*_ − *I*_*h*_|/Σ_*h*_Σ_*i*_*I*_*h,i*_, where *I*_*h*_ is the mean intensity of the *i* observations of symmetry-related reflections of *h. R* = Σ|*F*_obs_ − *F*_calc_|/Σ*F*_obs_, where *F*_calc_ is the calculated protein structure factor from the atomic model (*R*_free_ was calculated with 5% of the reflections selected)

**Fig. 4:**
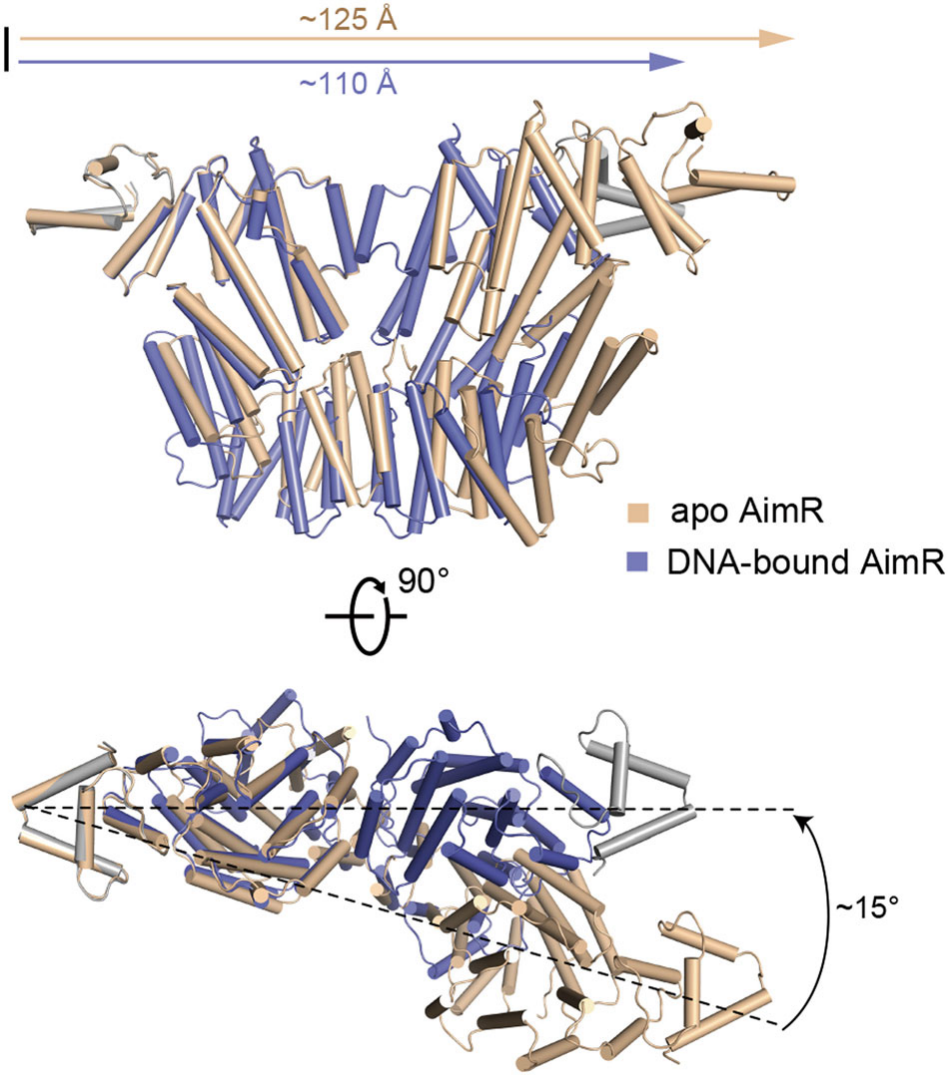
Structural alignment between the apo AimR and DNA-bound AimR. Conformational changes in AimR upon DNA-binding. The apo form of AimR is colored in wheat and DNA-bound AimR is colored in slate blue and gray

Interestingly, alignment of DNA-bound AimR with apo- or peptide-bound AimR revealed that the DNA-bound AimR exhibits a strongly closed conformation. The distance between the two aspartic acid residues in the inner cavity, D15 of AimR_A_ and D15 of AimR_B_, is approximately 125 Å in apo- or peptide-bound AimR. However, the distance between these two aspartic acid residues is nearly 110 Å in the AimR–DNA complex. A structure alteration also occurs at the C-terminal domain of AimR in the complex, with the side measurement changed to ~57 Å in the dimeric AimR (~50 Å in the apo form AimR). In addition, the DBD motif of AimR_B_ shows conformational variation, with a rotation of approximately 15° towards the protomer AimR_A_ (Fig. 4). These structural analyses suggest that AimR has distinct conformations, here, named the open and closed conformations, upon peptide or DNA binding.

### The arbitrium peptide stabilizes the AimR dimer in the open state

**Fig. 5:**
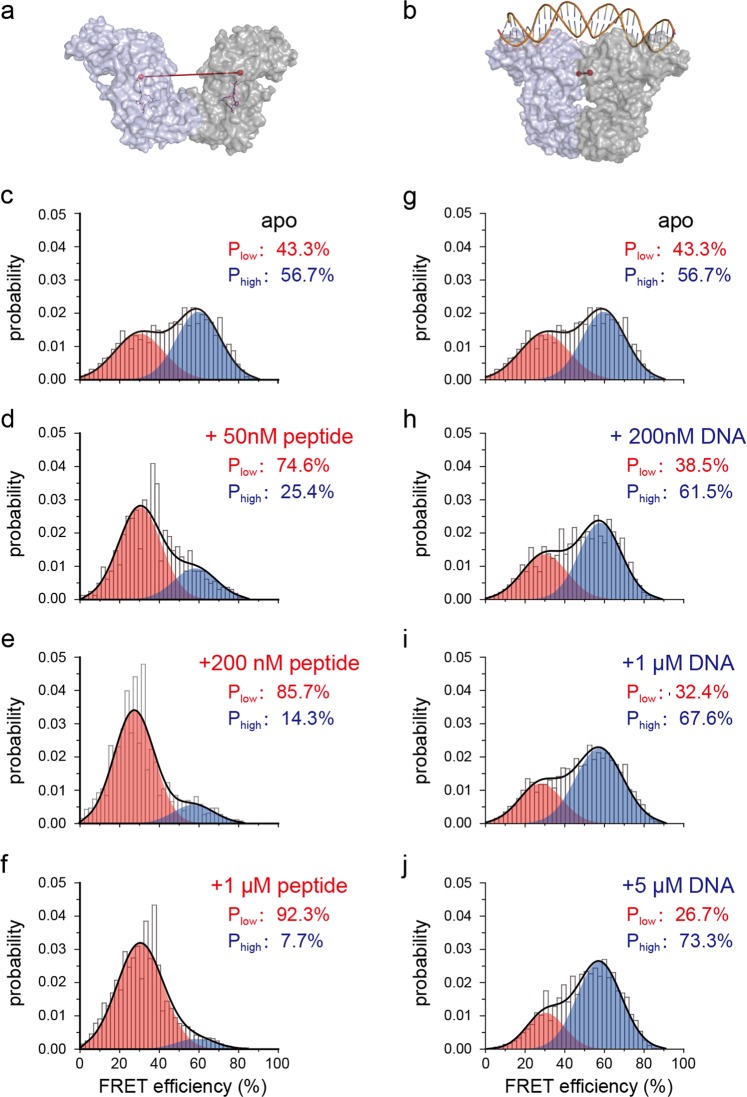
AimR adopts two conformational states for targets selectively recognition. **a**, **b** The distance between the fluorophores labeling sites in the peptide-bound AimR (red line) is longer than that in the DNA bound AimR (blue line), indicating low- and high-FRET efficiency, respectively. The Ca atoms of the Val129 are shown as red spheres, the peptide and the DNA are shown as sticks. **c**, **g** The smFRET profile (gray histogram) of AimR can be fitted as the sum of the two FRET species (black line). The low- and high-FRET species are colored red and blue, respectively. The arbitrium peptide selectively enriches the low-FRET species (**d**–**f**), and the DNA selectively enriches the high-FRET species (**h**–**j**). The FRET efficiency centers change little upon the ligand titrations. Note that panels (**c**) and (**g**) are identical in order to convenient tracking the population changes upon peptide or DNA binding

To characterize how the conformation of AimR changes in solution, we performed smFRET analysis. For fluorescent probe labeling, we mutated all six solvent-exposed cysteines to serines (C71S/C107S/C213S/C275S/C304S/C347S) and generated an additional V129C mutation to enable specific dye labeling through thiol-maleimide chemical conjugation (Fig. 5a, b). The smFRET data clearly showed that the FRET profile of AimR can be fitted to two FRET species. The low- and high-FRET efficiencies are centered at 30% and 60%, with respective populations of ~43% and ~57% (Fig. 5c, g). Hence, in solution, AimR alone exists in two dynamic conformational states that correspond to the open and closed conformations according to the low- and high-FRET efficiencies.

To assess the dynamic conformational states for target binding, we titrated the arbitrium peptide and the DNA into the solution and monitored the smFRET profile changes. The arbitrium peptide enriched the low-FRET species, while the high-FRET population was reduced (Fig. 5c–f). However, an unrelated peptide had no effects on the FRET profile (Supplementary Fig. S8d). In contrast, the high-FRET species were selectively enriched by the cognate DNA at the expense of the low-FRET species (Fig. 5g–j). Thus, the open conformation accounts for peptide binding, while the closed conformation accounts for DNA binding. Confirming this conclusion, the smFRET measured dye–dye distances in the two conformational states were consistent with the theoretically calculated distances in the crystal structures (Supplementary Fig. S9). The FRET species were selectively enriched with no apparent FRET efficiency shift during peptide or DNA titration, implying that AimR recognizes the arbitrium peptide and DNA via a conformational selection mechanism.

**Fig. 6:**
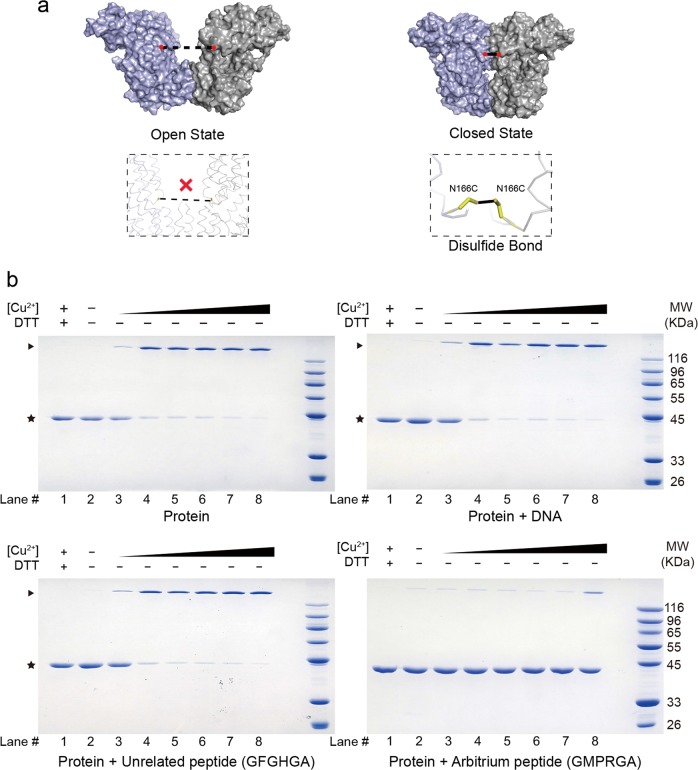
The arbitrium peptide stabilizes the AimR dimer in the open state. **a** Dimeric interface II is mediated by residues N166 in the closed state of AimR. Asn166 of each protomer was mutated to Cys for disulfide bond formation. Both residues are labeled red. **b** Crosslinking of AimR-N166C. Left upper panel: Crosslinking of AimR-N166C with increasing concentrations of o-phenanthroline copper complex (Cu^2+^). The reactants were subjected to nonreducing SDS-PAGE followed by Coomassie blue staining. Right upper panel: Crosslinking of AimR-N166C with increasing concentrations of Cu^2+^ in the presence of DNA. Left lower panel: Crosslinking of AimR-N166C with increasing concentrations of Cu^2+^ in the presence of an unrelated peptide. Right lower panel: Crosslinking of AimR-N166C with increasing concentrations of Cu^2+^ in the presence of the arbitrium peptide. The highest concentration of Cu^2+^ in lane 8 is 1 mM, and the concentration decreased sequentially by a 1:3 gradient from lane 8 to lane 3. The addition of 5 mM DTT effectively broke the disulfide bond (Lane 1)

To verify whether AimR indeed exists as two distinct states in solution, we employed a disulfide bond-mediated crosslinking strategy (Fig. 6a). Structural analysis showed that the distance between Asn166 of AimR_A_ and AimR_B_ is 3.5–4 Å in the closed conformation (Fig. 1b) but more than 25 Å in the open conformation. We generated a cysteine-substitution mutant (AimR N166C). Upon o-phenanthroline copper treatment, AimR showed a stable homodimer, indicating disulfide bond formation (Fig. 6b, left upper panel). The crosslinked AimR (N166C) and wild-type AimR showed similar behavior according to the nearly identical elution volumes on size-exclusion chromatography (Supplementary Fig. S10). In the presence of target DNA, AimR (N166C) also presented as a stable homodimer (Fig. 6b, right upper panel). However, AimR N166C could not be well crosslinked upon arbitrium peptide incubation, and the unrelated peptide had little effect. These results suggest that AimR exists in the closed and open conformations in solution and converts to the open conformation in the presence of the arbitrium peptide.

We hypothesized that the arbitrium peptide is able to stabilize AimR in the open state even after exposure to DNA. To test this scenario, we performed a completion experiment at the single-molecule level. Before binding saturation, 1 μM DNA increased the population of the high-FRET species of AimR from 56.7 to 67.6% (Fig. 5g, i). However, the addition of 1 μM arbitrium peptide dramatically increased the low-FRET species, while the high-FRET species was reduced to 6.1% (Supplementary Fig. S8a–c). These data suggested that the arbitrium peptide selectively enriches and stabilizes the preexisting open conformation of AimR, while the closed conformation is converted to the open conformation, leading to DNA dissociation.

## Discussion

**Fig. 7:**
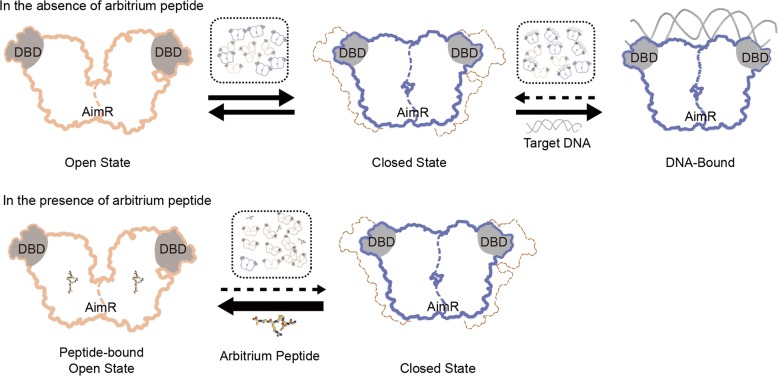
A working model of the arbitrium peptide. In the absence of the arbitrium peptide, dimeric AimR exhibits the dynamic open and closed conformations equally, which favor peptide and DNA binding, respectively. AimR is trapped in the open state and unable to bind DNA in the presence of the arbitrium peptide

In the phage arbitrium communication system, the AimR protein binds to the arbitrium peptide to lose its DNA-binding activity and subsequently regulates gene expression to determine the phage lysis–lysogeny transition^11–13^. Previously, we reported the structures of AimR from the SPbeta phage with and without peptide and clearly elucidated the specificity of peptide recognition, although the N-terminal DBD could not be well modeled^15^. Here, we updated the apo- and peptide-bound form structures containing the DBD, although there were only subtle differences between them (Supplementary Fig. S7d). How peptide binding diminishes the DNA targeting activity of AimR must be urgently answered. In this study, we determined the crystal structure of the AimR–DNA complex, in which AimR displays a strongly closed dimeric conformation. Using smFRET assay, we observed that in solution, AimR presents at least two major conformations: open and closed states that correspond to the two obtained structures (Fig. 5c, g). Importantly, when treated by DNA titration, the population of closed AimR increased, but most of the AimR was stabilized in the open conformation after saturation with the arbitrium peptide. Thus, we proposed a peptide regulation model for the SPbeta phage arbitrium communication system. In the absence of the arbitrium peptide, dimeric AimR exhibits the dynamic open and closed conformations equally, which favor peptide and DNA binding, respectively. Upon peptide binding, AimR is trapped in the open state, and it is difficult to transition to the closed state. In turn, the peptide inhibits AimR binding to the target DNA (Fig. 7). Notably, this conformational transition inhibitory mechanism is distinct to that in the phi3T phage. The phi3T peptide is likely to disrupt the dimeric state of the AimR protein and induce a shift to the monomeric state, diminishing its DNA-binding activity^11,16,18^. Interestingly, the apo phi3T AimR shows a structure similar to that of SPbeta DNA-bound AimR (Supplementary Fig. S11). This observation raised an intriguing question of whether the AimR family in phages has open and closed conformations. We endeavor to answer this question by dynamic approaches in the future.

As a quorum-sensing communication system in Gram-positive bacteria, RRNPP family regulators bind to signal peptides to become activated. The peptides regulate the RRNPP receptors through oligomer state transition or allosteric conformation changes. For example, binding of the peptide NprX promotes the conversion of dimeric NprR to a tetramer that specifically targets DNA to modulate gene expression^19^. In addition, the peptide cCF10 triggers PrgX tetramer-to-dimer dissociation and reduces the affinity of PrgX for the DNA-binding site of the targeted operon^20^. Alternatively, binding of the peptide PapR to PlcR leads to drastic conformational changes in the linker helix and N-terminal HTH domain and promotes the DNA binding of PlcR as a result^21,22^. Furthermore, the peptide XIP not only induces ComR dimerization but also releases the sequestered HTH domain to allow DNA binding during the activation process^23^. These crystal structures elucidated the regulatory mechanism of the peptides. Although the structural organization of phage AimR is reminiscent of the RRNPP family regulators, how the peptide regulates receptor activity is somewhat different. The proposed conformational transition inhibition mechanism here expands our understanding of the bacterial peptide communication pathway.

Only two DNA-bound complex structures have been reported in RRNPP family regulators, the PapR–PlcR–DNA complex and the XIP–ComR–DNA complex^21,23^. Here, we explored a third case concerning the structure of DNA-bound AimR, which contains a palindromic sequence (5′ ACT……AGT 3′) within a 31-bp DNA fragment. On a structural basis, AimR binds to DNA as a dimer with helix α3′ of the N-terminal DBD and employs the side chains of Asn30 and Asn32 to make base-specific contacts with bases T31, A29′, and G30′ of the palindromic sequence. Additional nonspecific contacts between some positive amino acids of AimR and the phosphate backbone of DNA further stabilize the complex. Nevertheless, mutagenesis of the palindromic sequence was unable to completely disrupt AimR DNA binding activity, as substitution mutants at position 3 of the palindromic sequence showed activity comparable to that of the wild-type DNA (Fig. 2c). This finding raises the possibility that AimR targets similar sequences within the *Bacillus* genome, which hierarchically regulate the phage lysis process. The latest research explored other putative DNA targets of AimR in vitro, but the available information is still limited^24^. The regulation of multiple genes by AimR during the lysis–lysogeny transition needs to be further investigated in vivo.

## Materials and methods

### Bacterial strains, plasmids, and phage

*B. subtilis* strain 168 (BGSCID: 1A1) and *B. subtilis* strain CU1065 (BGSCID: 1A100) were obtained from the Bacillus Genetic Stock Center (BGSC). Wild-type and mutant *aimR* genes with their own promoters were cloned into the pDG1730 vector. These plasmids were transformed into *B. subtilis* CU1065 and integrated into the *amyE* locus of the *B. subtilis* genome, as previously described^25^. Phage SPbeta was induced from *B. subtilis* strain 168 using 0.5 μg/ml mitomycin C (MedChemExpress, HY-13316) for 3 h and was then purified and amplified in *B. subtilis* strain CU1065^26^. The *aimR* deletion phage strain was generated as described previously^15^.

### Protein preparation

The codon-optimized complementary DNA of full-length *aimR* from SPbeta (GenBank: NC_001884) was subcloned into the pET21b vector (Invitrogen) with a C-terminal 8× His tag. The AimR clone was transformed into *Escherichia coli* strain BL21 (DE3) and induced with 0.2 mM isopropyl β-D-thiogalactopyranoside at an absorbency (600 nm) of 1.1. After induction at 16 °C for 16 h, the cells were collected and resuspended in buffer containing 25 mM Tris–HCl (pH 8.0) and 150 mM NaCl. Following further disruption by a homogenizer (JNBIO), cell debris was removed via centrifugation at 23,000*g* for 1 h. The supernatant was collected and loaded onto Ni^2+^-nitrilotriacetate affinity resin (Ni-NTA, Qiagen), followed by washing with 25 mM Tris–HCl (pH 8.0), 150 mM NaCl, and 20 mM imidazole. The AimR protein was eluted using a buffer containing 25 mM Tris–HCl (pH 8.0) and 250 mM imidazole. The eluted protein was further purified using anion-exchange chromatography (Source 15Q 10/100, GE Healthcare). The elution peak was subjected to size-exclusion chromatography (Superdex-200 Increase 10/300, GE Healthcare) in a buffer containing 25 mM Tris–HCl (pH 8.0), 150 mM NaCl, and 5 mM dithiothreitol (DTT). The peak fractions were collected for crystallization. The AimR mutants were generated using a two-step PCR strategy and were subcloned into pET15b, overexpressed and purified in the same way as the wild-type protein. Following proteolytic removal of the hexahistidine (6× His) tag by drICE, the mutation proteins were purified using size-exclusion chromatography in a buffer containing 25 mM Tris–HCl (pH 8.0) and 150 mM NaCl for biochemical assays.

### Crystallization

For the apo form and peptide-bound AimR, the purified protein was concentrated to ~270 µM (~12.5 mg ml^−1^), incubated on ice for 30 min with or without the arbitrium peptide GMPRGA (2.7 mM) (GLS Biochem). For the DNA-bound AimR, the purified protein was concentrated to ~270 µM (~ 12.5 mg ml^−1^), incubated on ice for 30 min with 276 µM 31 bp DNA fragment (77710–77740, 5′ ACTTAAATATTAGGTTTTAATAACATCTAGT 3′) and 5 mM MgCl_2_. All sample crystallized in hanging drops at 18 ℃ using a mixture of 1 µl of the sample and 1 µl of the crystallization buffer. Crystals of the apo form under the conditions in the presence of 0.1 M sodium cacodylate (pH 6.6), 11% polyethylene glycol 8000, and 0.2 M sodium chloride. The crystals of GMPRGA peptide-AimR under the 0.1 M sodium cacodylate (pH 6.1), 9% polyethylene glycol 8000, 0.2 M NaBr, and 5 mM DTT. The crystal of DNA-bound AimR was grown in 0.1 M sodium cacodylate (pH 5.8) and 11% polyethylene glycol 4000. The crystals grew to full size in 3 or 4 days. They were flash-frozen in liquid nitrogen and cryoprotected with well buffer and 60% glycerol mixture (v:v = 1:1), but the DNA binding form crystals were cryoprotected mixed with 40% glycerol (v:v = 1:1).

### Data collection and structure determination

All of the diffraction data were collected at the Shanghai Synchrotron Research Facility (SSRF) on beamline BL17U1 or BL19U1 and then integrated and processed using the XDS program^27–29^. Further data processing was performed using the CCP4 suite^30^. The structure of DNA-bound AimR was determined through molecular replacement with the apo-form AimR (PDB ID:5XYB) as the search model using the program PHASER^31^. The DNA-bound AimR structures were iteratively built using COOT and refined using the PHENIX program^32,33^. It is clear that a well-built model can be generated from the electron density of the AimR N-terminal residues 1–40. As we described, the diffraction data of apo-form AimR and peptide-bound form AimR were newly collected. Using NTD of DNA-bound AimR as the initial model, the N-terminal models of Apo-form AimR and peptide-bound AimR can be built. Similarly, COOT and PHENIX were applied to structural refinement. Data collection and structure refinement statistics are summarized in Table 1. All of the figures were generated using PyMOL (http://www.pymol.org/).

### Electrophoretic mobility shift assay

DNA probes were annealed using boiling water with FAM-labeled primers to generate the 37 bp DNA fragment (77707–77743, 5′ ATCACTTAAATATTAGGTTTTAATAACATCTAGTGAT 3′). The labeled probes (5 nM) were incubated with 0.004, 0.02, 0.1, and 0.5 μM AimR proteins or peptide-bound AimR complex (molar ratio = 10:1) in a buffer containing 25 mM Tris–HCl (pH 8.0), 5 mM MgCl_2_, 5 mM DTT, 0.1 mg ml^−1^ BSA, 10% glycerol, 150 mM NaCl, and 50 μg ml^−1^ salmon sperm DNA at 4 °C for 15 min. The reactions were resolved on 8% native acrylamide gels (37.5:1 acrylamide:bis-acrylamide) in 0.5× Tris–Boric acid buffer at 150 V for 3 h. Images of the gels were obtained using FLA5100 (Typhoon, Fuji, Japan).

### Growth dynamics of SPbeta-infected *B. subtilis*

Overnight cultures of *B. subtilis* CU1065 and *aimR*-overexpressing strains were diluted 1:100 in LB medium and incubated at 28 °C with shaking until the suspension reached OD_600_ = 0.1. The bacteria were infected with the SPbeta phage or the SPbeta Δ*aimR* phage at MOI = 0.001. The OD_600_ was measured every 30 min using a spectrophotometer (Jingke Instrument, 722N). All growth dynamics curves were drawn using Origin 8.0.

### Fluorescent dye conjugation

The point mutation of AimR-V129C (C71S/C107S/C213S/C275S/C304S/C347S/V129C) was generated with a standard PCR-based strategy and subcloned, overexpressed, and purified in the same way as the mutant proteins. The distance between two Ca atoms of V129 residues in peptide-bound AimR complex is 61.9 Å and is shorten to 30.8 Å in DNA-bound AimR complex that would produce discernible FRET efficiencies. Alexa Fluor 488 C_5_ maleimide (Thermo Fisher, A10254) and Cy5 maleimide (GE Healthcare, PA15131) (*R*_0_ = 52 Å^34^) were freshly dissolved in DMSO at 1 mM concentration before conjugation. A desalting column (HiPrep 26/10, GE Healthcare) was used to exchange the buffer of AimR V129C containing 20 mM phosphate, pH 7.4 and 100 mM NaCl, and the protein fraction was collected in premixed Alexa488 and Cy5 dye. The molar ratio of the conjugation reaction of AimR V129C:Alexa488:Cy5 was 1:4:6. Conjugation was performed for 3 h at room temperature in the dark, and the mixture was then purified by a Source-Q column (GE Healthcare). The fraction of double-labeled protein that exhibited absorption at 280, 493, and 640 nm was simultaneously collected for smFRET data collection.

### Single-molecule FRET data collection and analysis

The smFRET data collection and analysis were performed as previously described^35^. In brief, an A1 confocal microscope (Nikon, Japan) coupled to two picosecond pulsed diode laser heads (LDH-P-C-485B and LDH-P-C-640B, PicoQuant, Germany) and two SPCM-AQRH detectors (Excelitas, Canada) was used for fluorescence excitation and emission detection. A 60× water immersion objective (WI 60×, NA 1.20, Nikon, Japan) was used for confocal laser microscopy, and the pinhole size was set at approximately 100 μm. The laser power before the objective was set at ~100 μW for the 485-nm laser and ~35 μW for the 640-nm laser. Donor emission was filtered with an ET550/50 m bandpass (Chroma), and acceptor emission was filtered with an ET700/75 m bandpass (Chroma), before being focused onto the two SPCM-AQRH detectors. A pulsed interleaved excitation (PIE) scheme at a repetition of 32 MHz was employed for data collection to exclude the emitted photons that resulted from donor only or acceptor only species^36^.

The smFRET measurements were performed at 25 °C in pH 7.4 buffer containing 20 mM phosphate, 100 mM NaCl, and 0.01% (v/v) Tween 20, with additional 1 mM ascorbic acid and 1 mM methylviologen for photobleaching and blinking minimization^37^. A concentration of ~150 pM of dye-labeled protein was used for data collection. The arbitrium peptide (GMPRGA, GLS Biochem), an unrelated peptide (GFGHA, GLS Biochem) and 37 bp DNA (77707–77743, 5′ ATCACTTAAATATTAGGTTTTAATAACATCTAGTGAT 3′) were prepared as stock solutions in a buffer containing 20 mM phosphate, pH 7.4 and 100 mM NaCl and were mixed with AimR V129C prior to each experiment to achieve the desired concentrations. The smFRET data were typically collected for ~1 h. Photon time traces were binned with a 1 ms width, and 4–9 counts/bin were used as the threshold for burst searching. The burst searching process was performed using a handwritten script, and a minimum of 25 total photon counts was defined as a burst event. The exact FRET efficiencies were calculated based on our calibrated parameters for the instrument and fluorophores, and the FRET efficiency distribution was analyzed with a multi-Gaussian mixture using a handwritten script^35^.

### Intermolecular crosslinking of AimR

The point mutation AimR-N166C (C71S/C107S/C213S/C275S/C304S/C347S/N166C) was generated with a standard PCR-based strategy and subcloned, overexpressed, and purified in the same way as the mutant protein. After gel filtration, the peak fractions were collected for further characterization. The intermolecular crosslinking of AimR, targeting Cys166 in two adjacent protomers, was catalyzed by 2 mM freshly prepared o-phenanthroline copper complex^38,39^. The AimR-N166C protein at approximately 1 mg/ml was mixed with the GMPRGA peptide and DNA for 15 min. Then, these components were mixed with catalyst at the indicated concentrations and incubated at room temperature for 30 min. Reducing reagents were avoided in each step. The same amount of protein solution was incubated with 5 mM DTT at room temperature for 30 min as a control. For the EMSA assay, the mutant protein was treated with 2 mM o-phenanthroline copper complex and further purified through gel filtration (Superdex-200 10/300, GE Healthcare) in buffer containing 25 mM Tris–HCl (pH 8.0) and 150 mM NaCl.

## Supplementary information

supplementary information.

## Data Availability

Atomic coordinates and structure factors of the apo form AimR, peptide-bound form AimR, and DNA-bound form AimR have been deposited to Protein Data Bank under accession numbers 6JG5, 6JG9, and 6JG8, respectively.

## Supplementary information

**Supplementary Information** accompanies the paper at (10.1038/s41421-019-0101-2).
