# Supplementary material for: Structural insights into DNA recognition by AimR of the arbitrium communication system in the SPbeta phage
Source: Cell Discov. 2019 May 28;5:29. doi: 10.1038/s41421-019-0101-2 (PMC6536502; doi:10.1038/s41421-019-0101-2)
Supplement: Supplementary file 1 — supplementary information. [file 41421_2019_101_MOESM1_ESM.docx]

**SUPPLEMENTAL INFORMATION**

**Structural insights into DNA recognition** **by AimR of the arbitrium communication system in the SPbeta phage**

Zeyuan Guan^1, †^, Kai Pei^1, †^, Jing Wang^1^, Yongqing Cui^1^, Xiang Zhu^1^, Xiang Su^1^, Yuanbao Zhou^1^, Delin Zhang^1^, Chun Tang^2^, Ping Yin^1^, Zhu Liu^1^, Tingting Zou^3^^,*^

^1^ National Key Laboratory of Crop Genetic Improvement and National Centre of Plant Gene Research, Huazhong Agricultural University, Wuhan, Hubei 430070, China

^2^ CAS Key Laboratory of Magnetic Resonance in Biological Systems, State Key Laboratory of Magnetic Resonance and Atomic Molecular Physics, and National Center for Magnetic Resonance at Wuhan, Wuhan Institute of Physics and Mathematics of the Chinese Academy of Sciences, Wuhan, Hubei Province 430071, China.

^3^ College of Life Science and Technology, Huazhong Agricultural University, Wuhan, Hubei 430070, China

^†^These authors contributed equally to this work as first authors.

*To whom correspondence should be addressed. Tel: (+86) 27 87288920; Email: [zoutingting@mail.hzau.edu.cn](mailto:zoutingting@mail.hzau.edu.cn)


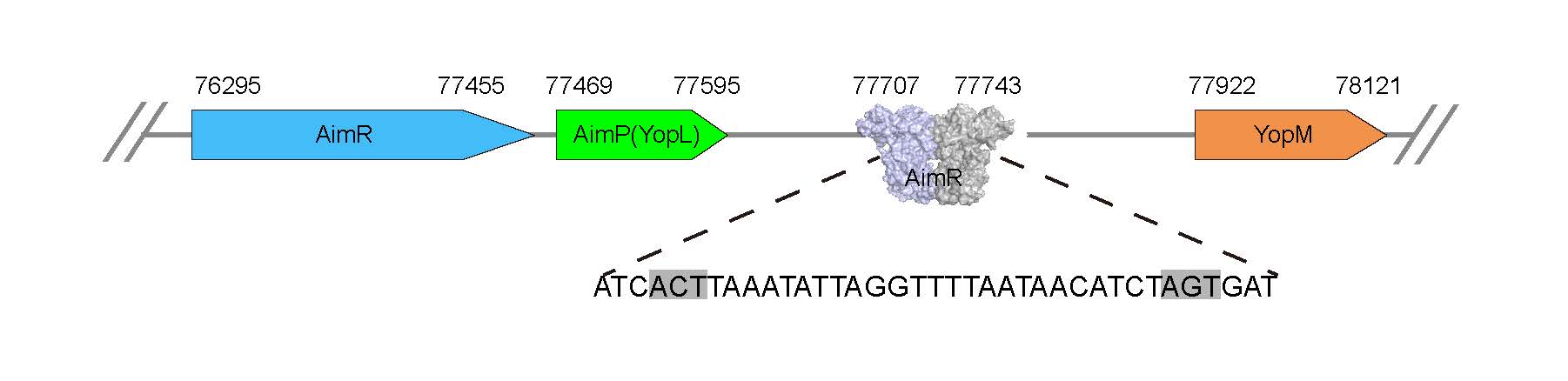


**Supplementary Fig. S1** AimR binds to DNA by recognizing a palindromic sequence (5′ ACTTAAATATTAGGTTTTAATAACATCTAGT 3′, locus 77710-77740) downstream of the *aimP* gene in the genome of the SPbeta phage.


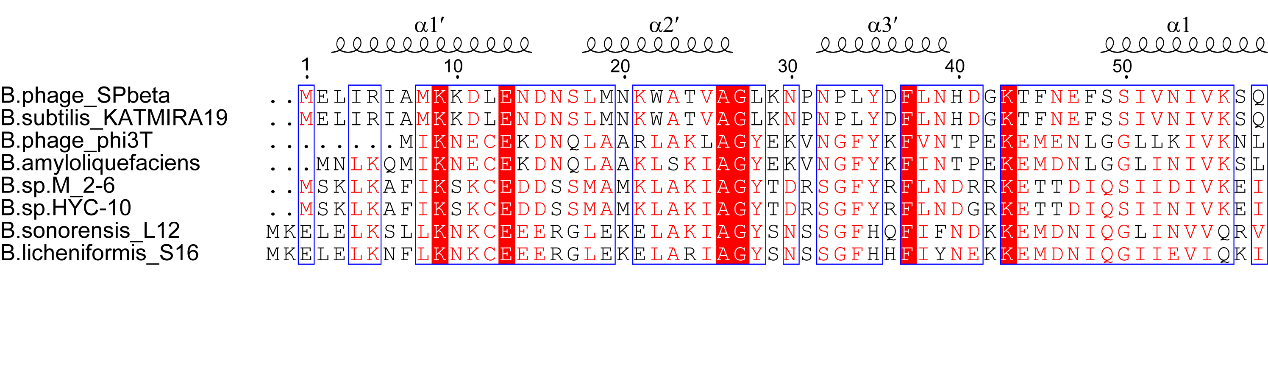


**Supplementary Fig. S2** Multiple sequence alignment (generated by Multalin) displays N-terminal sequences of AimRs from the *Bacillus* phage SPbeta (NCBI Reference Sequence: NP_046633.1) and seven homologues: *Bacillus subtilis* KATMIRA1933 (GenBank: KDE21692.1), *Bacillus* phage_phi3T (GenBank: APD21232.1), *Bacillus amyloliquefaciens plantarum* YAU B9601-Y2 (NCBI Reference Sequence: WP_014417907.1), *Bacillus* sp. M_2-6 (GenBank: EIL85391.1), *Bacillus* sp. HYC-10 (GenBank: EKF35089.1), *Bacillus sonorensis* L12 (GenBank: EME72281.1) and *Bacillus licheniformis* S16 (GenBank: EWH19854.1). Secondary structural elements of the DBD are indicated above the sequences.


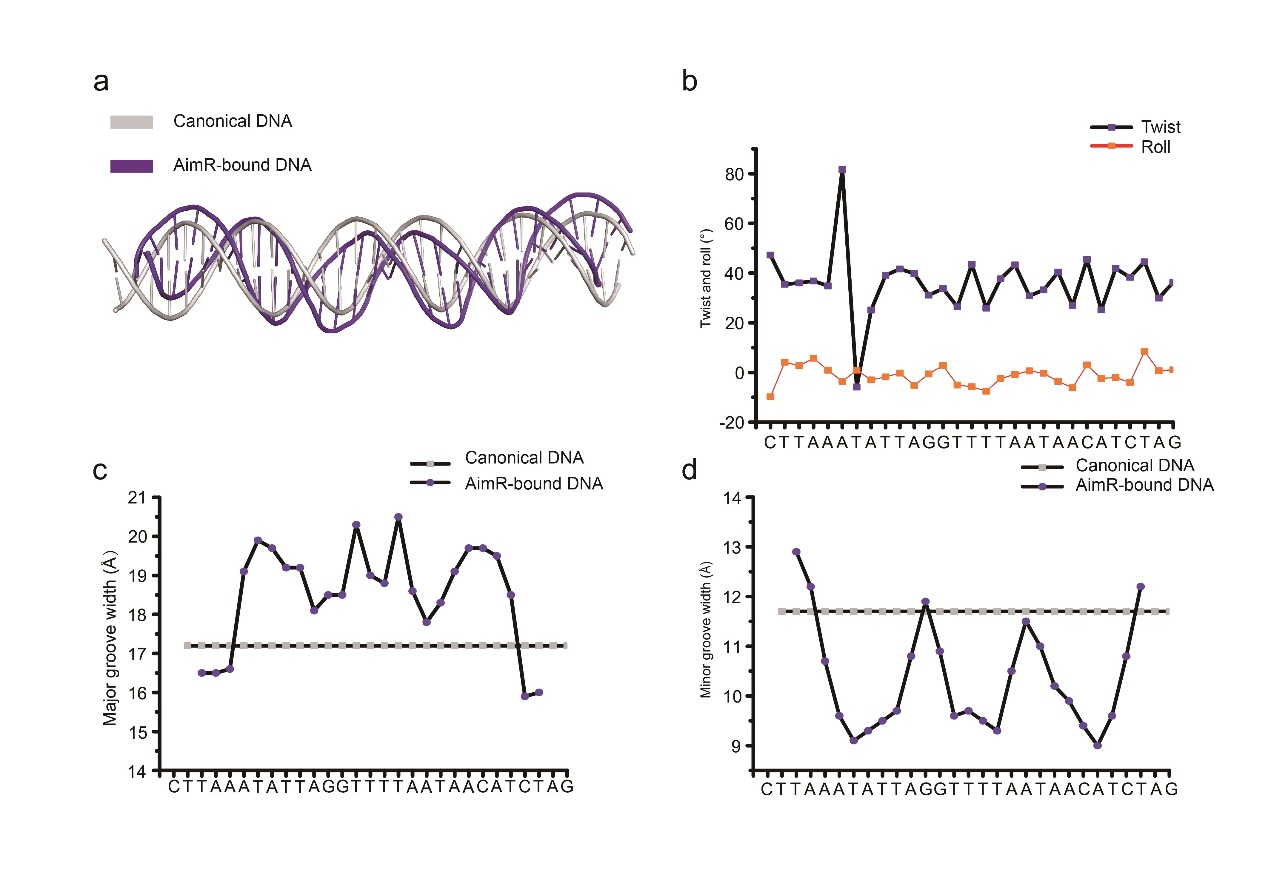


**Supplementary Fig. S3** DNA deformation in the AimR-DNA complex. **a** Structural comparison of the AimR-bound DNA with a canonical B-DNA double helix. AimR-bound DNA is highlighted in purple, while canonical B-DNA is shown in gray. **b** The roll and twist angles for each base pair step of the AimR-bound DNA. **c** The major groove width of the AimR-bound DNA and canonical B-DNA. **d** The minor groove width of the AimR-bound DNA and canonical B-DNA.


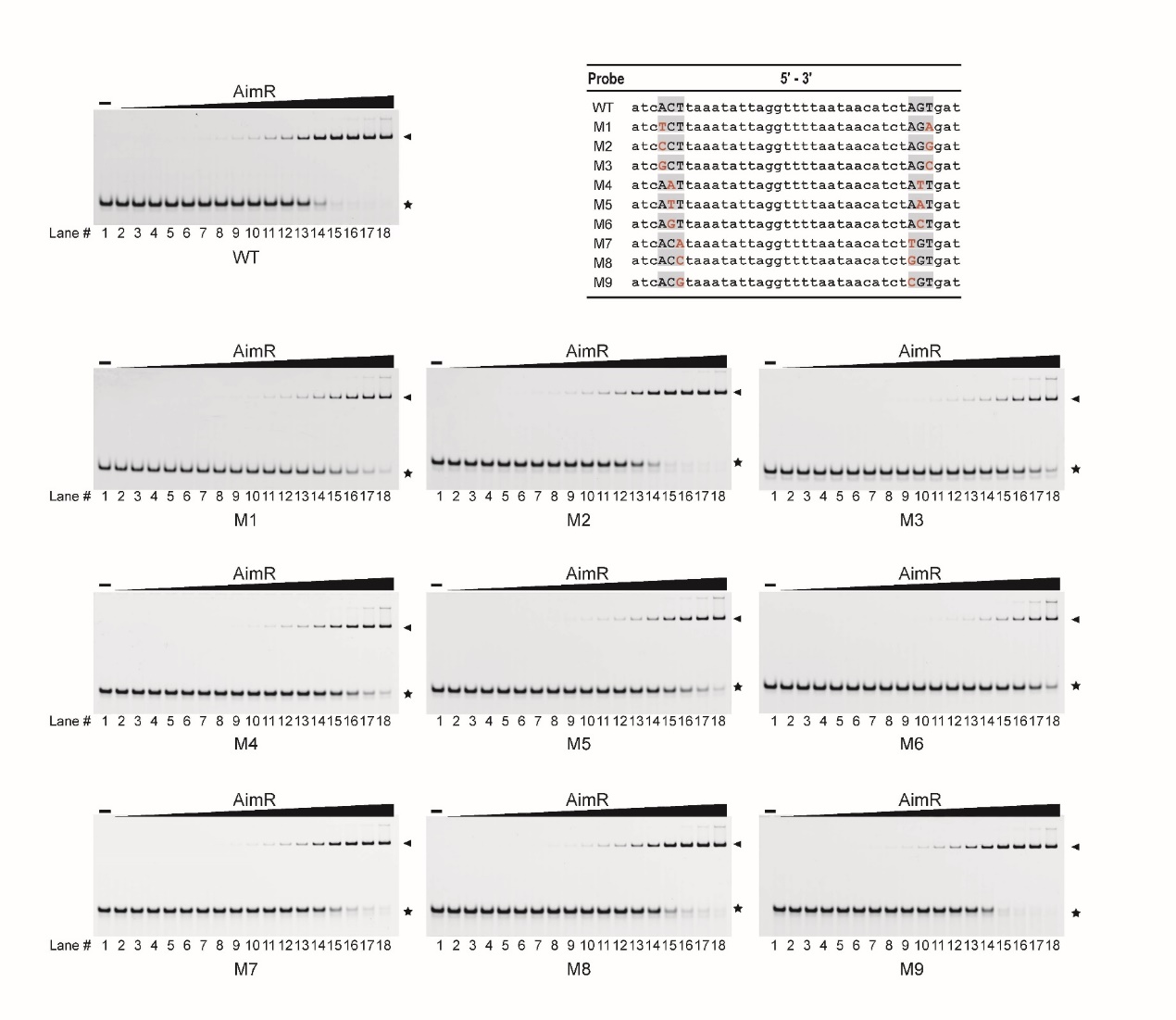


**Supplementary Fig. S4** The binding affinities between AimR and DNA mutants. Interactions of AimR and mutant DNA fragments were examined via EMSA. The DNA concentration was 20 nM, and the protein gradient concentrations in lanes 1~5 were 0, 0.008, 0.04, 0.2, and 1 μM. Star represents free probe, and black triangles represent shifted bands. The experiments were repeated twice, and a representative result is shown. DNA probes are listed in the table. The mutated bases are highlighted in red. Quantification of these data is listed in Fig. 2c.


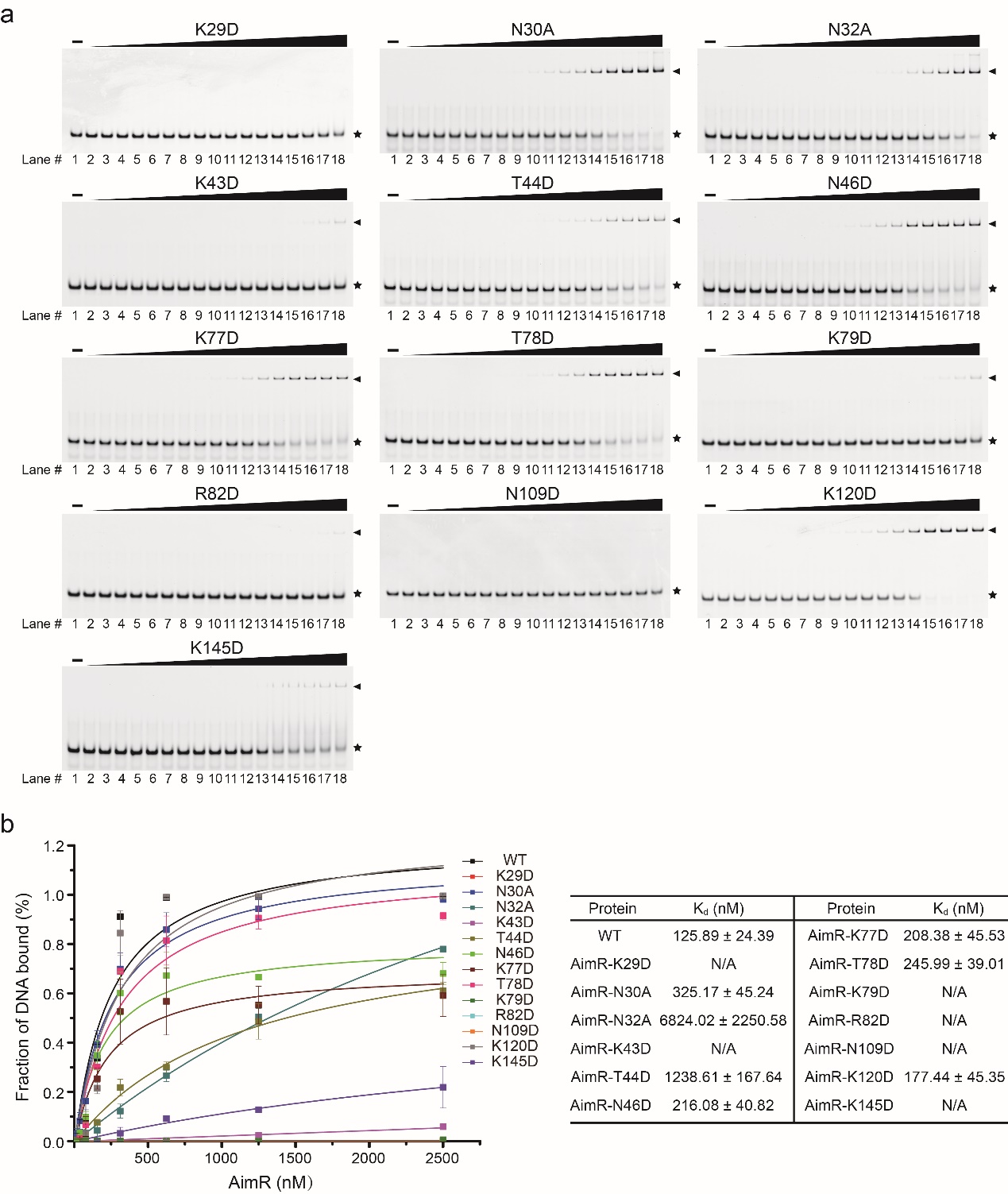


**Supplementary Fig. S5** The DNA-binding affinities of AimR proteins. **a** The DNA-binding affinities of AimR mutants were measured by EMSA. The highest concentration of protein in lane 18 is 0.5 μM, and the concentration decreased sequentially by a 1:2 gradient from lane 18 to lane 2. Complex formation is indicated by a triangle, and free DNA is indicated by a black asterisk. **b** The DNA-binding activities of AimR proteins and the calculated *K*_d_ values are indicated. These experiments were performed three times with equivalent results. *K*_d_ values are presented as the mean ± s.d.


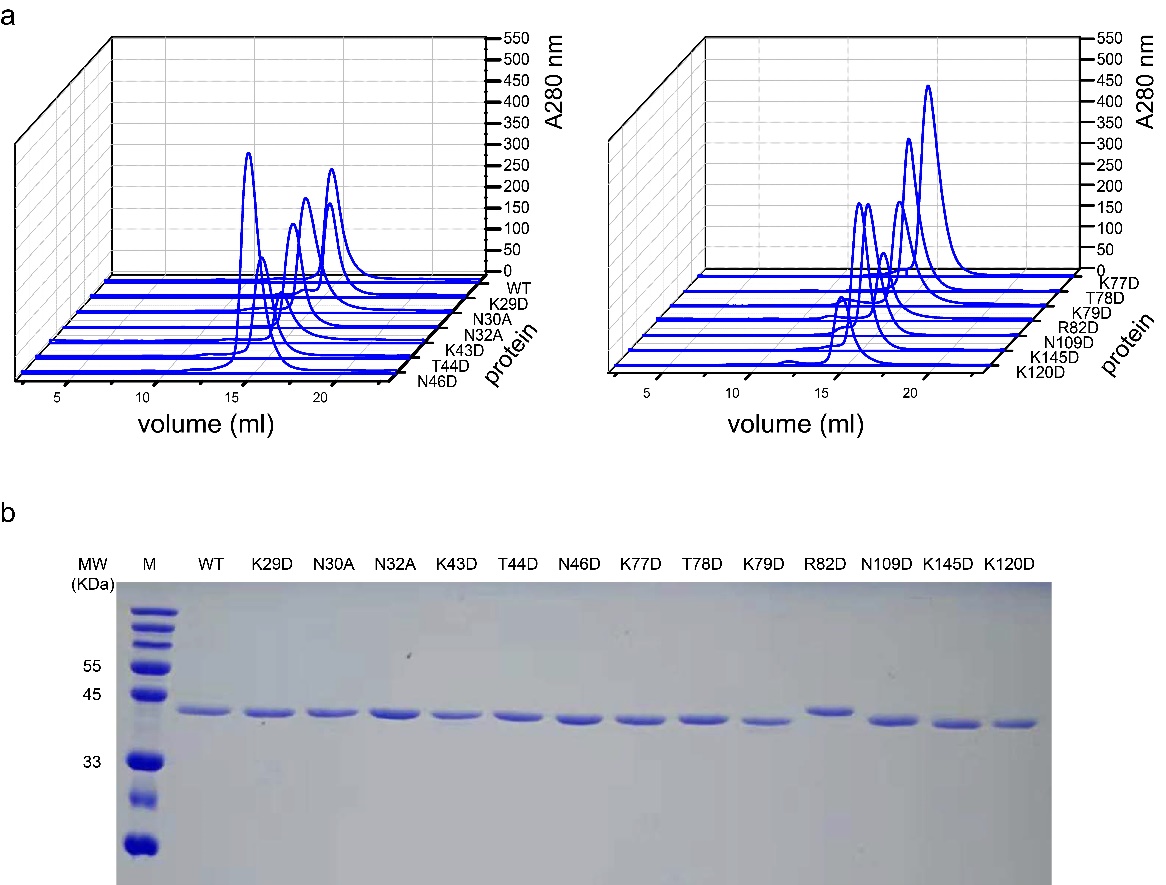


**Supplementary Fig. S6** Purification of AimR mutants. **a** Purified AimR mutants were subjected to gel filtration via Superdex 200. **b** The peak fractions eluted at 15 ml were analyzed by SDS-PAGE.


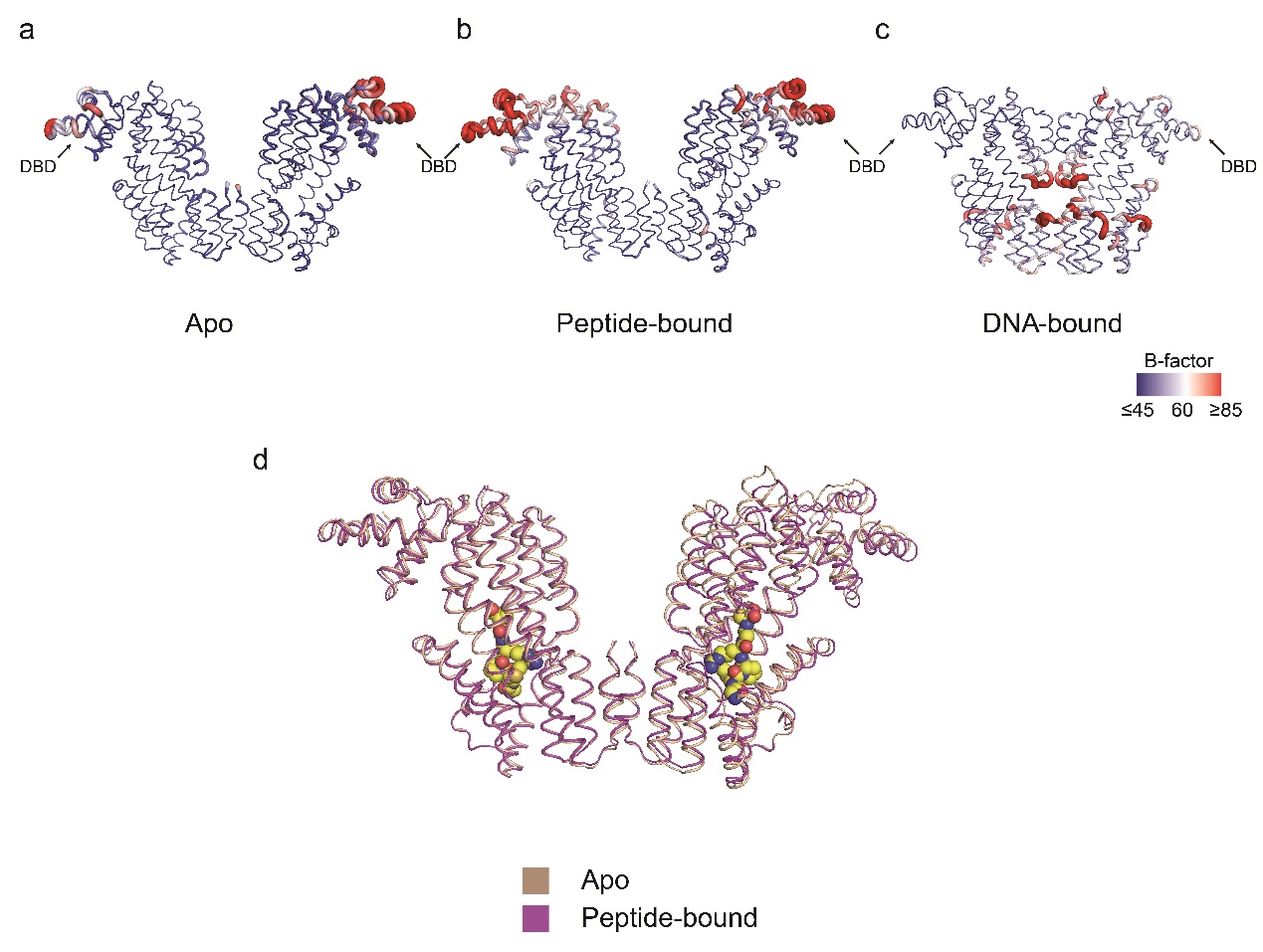


**Supplementary Fig. S7** Crystallographic B factors of AimR in apo, peptide-bound and DNA-bound forms. **a** Apo-structure of AimR. **b** Arbitrium peptide-bound structure of AimR. **c** DNA-bound structure of AimR. Crystallographic B factors are indicated by color, from blue to red, and via a putty tube representation. **d** Conformational change of AimR upon arbitrium peptide binding. The AimR structure in the apo form (wheat) is superimposed with that in the GMPRGA peptide-bound form (magenta).
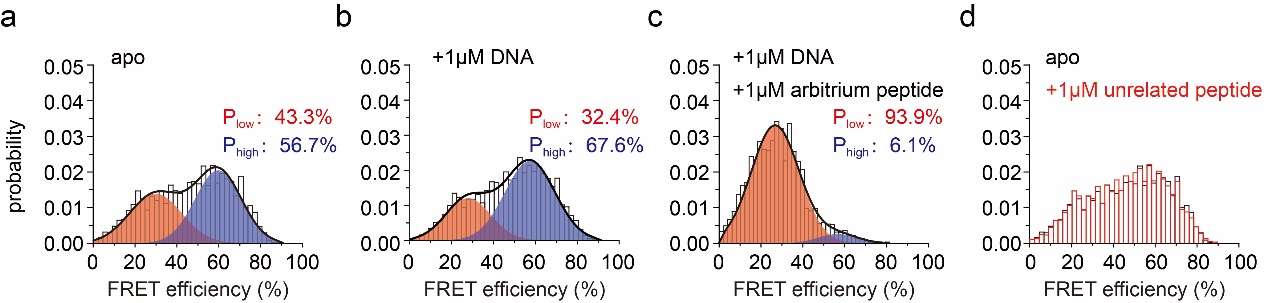


**Supplementary Fig. S8** Arbitrium peptide antagonize the interaction between DNA and AimR. **a** smFRET profile can be fitted to two FRET species, with the low-FRET species of about 43% population and the high-FRET species of about 57% population (Fig. 5c, g). **b** 1μM DNA enriches the high-FRET species indicating that the DNA selectively recognizes the pre-existing closed conformation of AimR (Fig. 5i). **c** smFRET competition experiment, with 1μM DNA and 1μM arbitrium peptide presented simultaneously, the low-FRET species is increased to about 94% while the high-FRET species down to about 6%. The data indicate that the AimR prefers bind peptide to DNA. **d** An unrelated peptide (GFGHGA) has little perturb on the AimR FRET profile indicating no interactions.


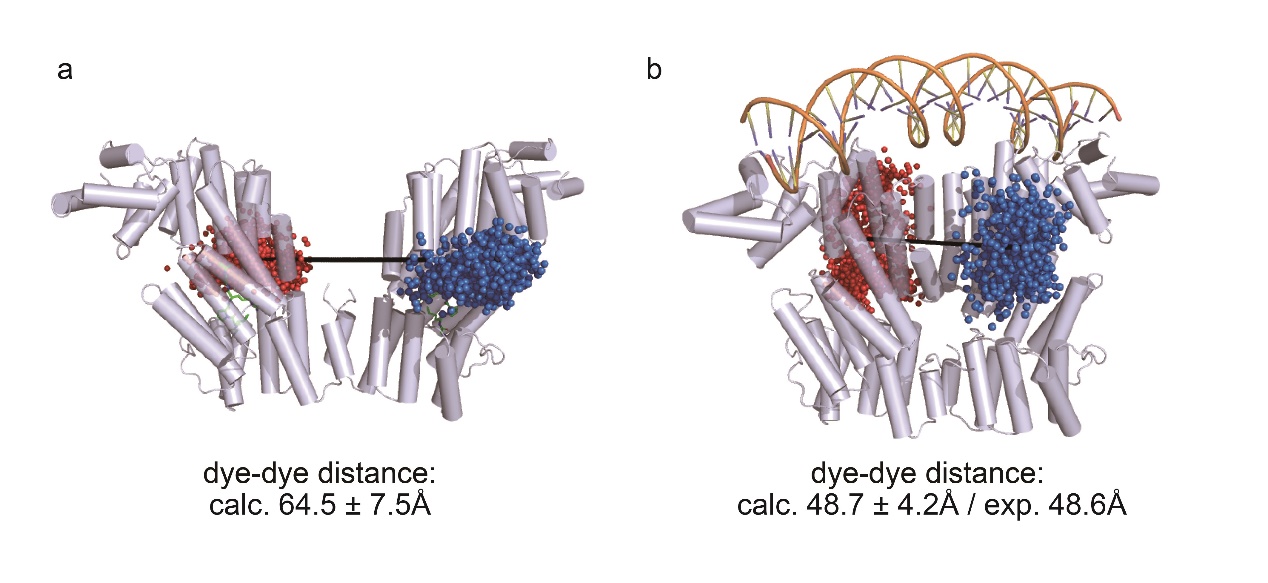


**Supplementary Fig. S9** The calculated average distances are consistent with smFRET data. The fluorophores are patched on the Val129C using Xplor-NIH^1^, and the linker between the protein and the rigid portion of the fluorophores are given torsion angle freedom and are allowed reorient. The FRET distance can be calculated between the mass center of Alexa 488 (red spheres) and the Cy5 (blue spheres) based on the crystal structures (cartoon). The calculated average distance of the apo AimR is 64.5 ± 7.5Å, consisting with the smFRET measured 60.0 Å of the low-FRET species (**a**), and the calculated average distance of the DNA bound AimR is 48.7 ± 4.2Å, consisting with the smFRET measured 48.6 Å of the high-FRET species (**b**).


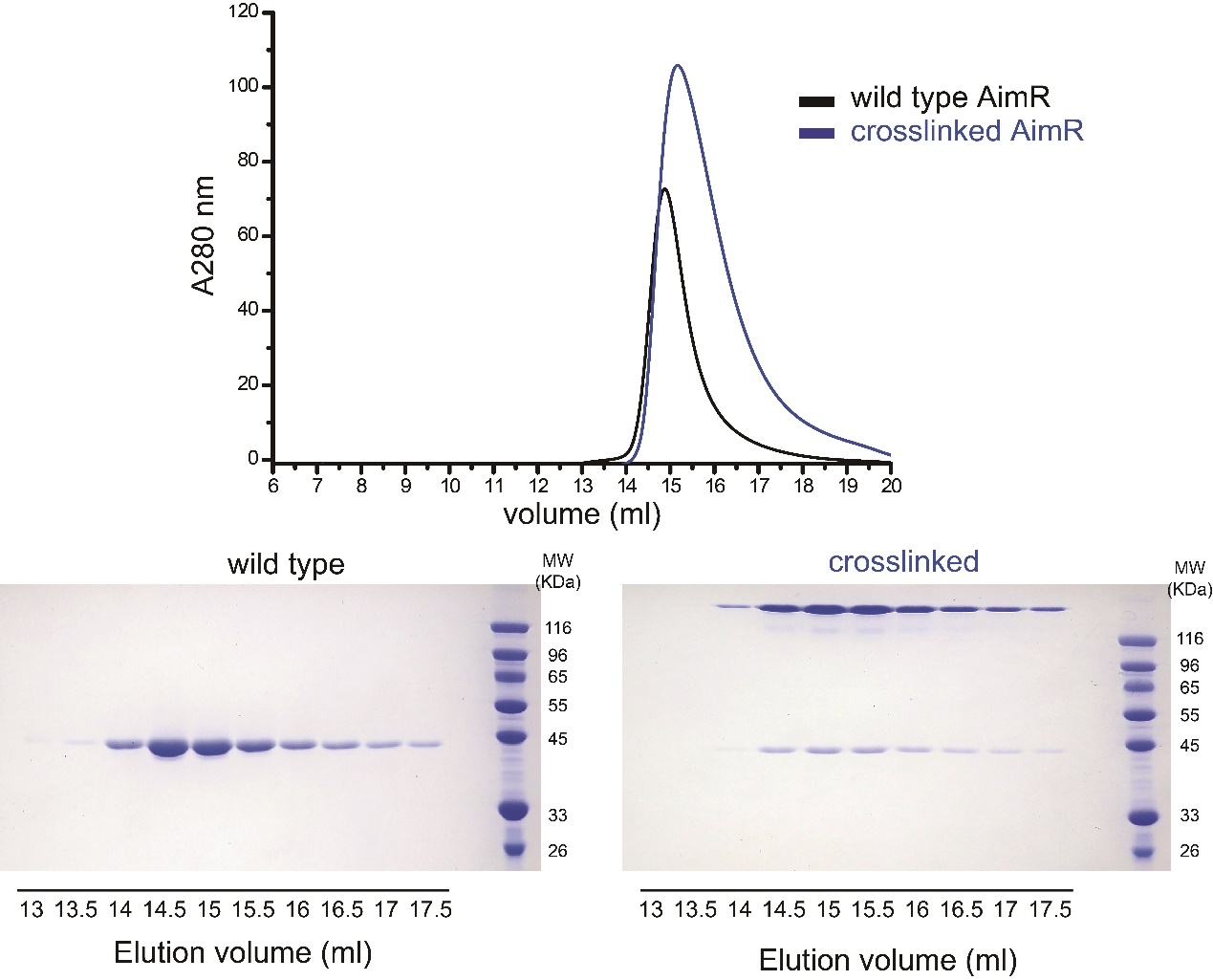


**Supplementary Fig. S10** Crosslinked AimR exhibits similar solution behavior to that of wild-type AimR. Wild-type AimR (black line, 14.90 ml) and crosslinked AimR (blue line, 15.14 ml) are both in the dimer state.


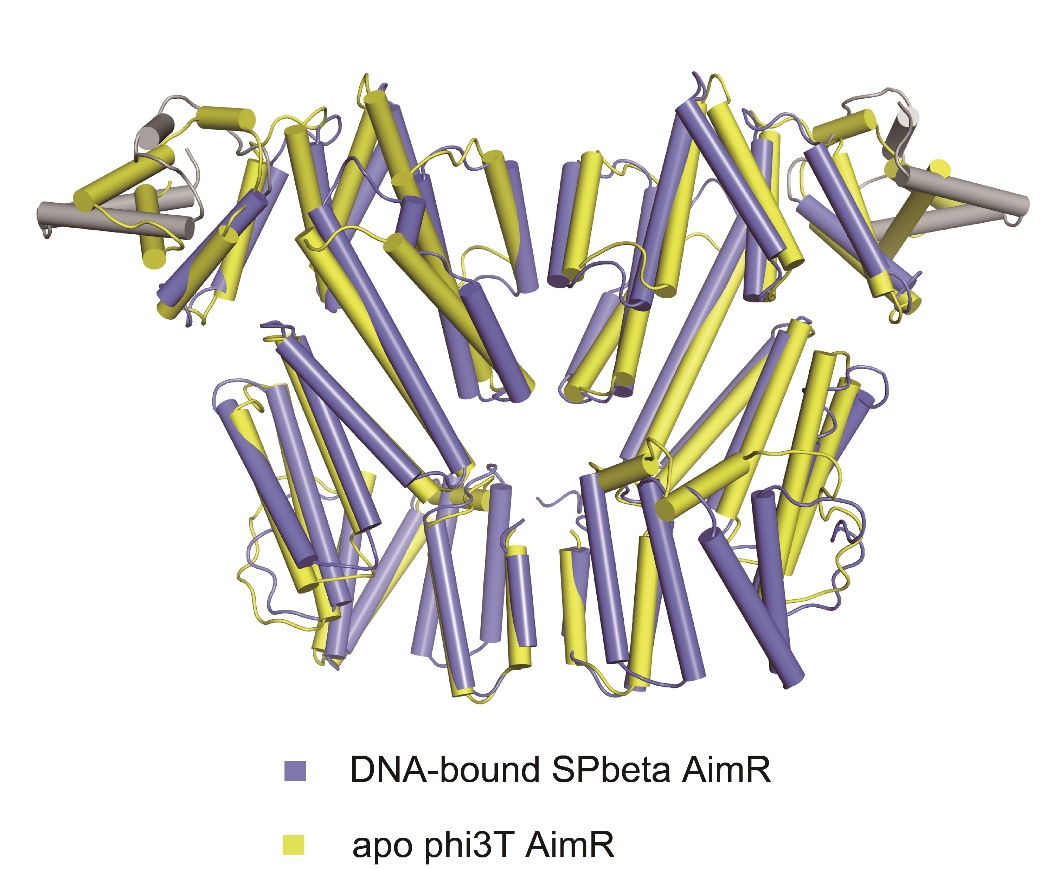


**Supplementary Fig. S11** The phi3T AimR structure in the apo form (yellow) is similar to the DNA-bound SPbeta AimR (slate blue).

**REFERENCES**

1 Schwieters, C. D., Kuszewski, J. J. & Clore, G. M. Using Xplor-NIH for NMR molecular structure determination. *Prog Nucl Mag Res Sp* **48**, 47-62, doi:10.1016/j.pnmrs.2005.10.001 (2006).
